# Zebrafish as a model system for studying reproductive diseases

**DOI:** 10.3389/fcell.2024.1481634

**Published:** 2024-12-23

**Authors:** Wenwen Zha, Weitao Hu, Chenkai Ge, Jianjun Chen, Zigang Cao

**Affiliations:** ^1^ Jiangxi Engineering Laboratory of Zebrafish Modeling and Drug Screening for Human Diseases, Jiangxi Key Laboratory of Developmental Biology of Organs and Epigenetics, College of Chinese Medicine, Clinical Research Center of Affiliated Hospital of Jinggangshan University, Jinggangshan University, Ji’an, China; ^2^ Jiangxi Engineering Laboratory of Zebrafish Modeling and Drug Screening for Human Diseases, Jiangxi Key Laboratory of Organs Development and Epigenetics, Key Laboratory of Jiangxi Province for Biological Invasion and Biosecurity, College of Life Sciences, Clinical Research Center of Affiliated Hospital of Jinggangshan University, Jinggangshan University, Ji’an, China; ^3^ Shanghai Key Laboratory of Anesthesiology and Brain Functional Modulation, Clinical Research Center for Anesthesiology and Perioperative Medicine, Translational Research Institute of Brain and Brain-Like Intelligence, Department of Pediatrics, Shanghai Fourth People’s Hospital, School of Medicine, Tongji University, Shanghai, China; ^4^ Department of Big Data in Health Science School of Public Health, Institute of Medical Genetics, Tongji University School of Medicine, Tongji University, Shanghai, China

**Keywords:** reproductive diseases, zebrafish, germ cells, CRISPR, animal models

## Abstract

Reproductive system diseases have become a major health challenge facing humans, so extensive investigations are needed to understand their complex pathogenesis and summarize effective treatments. In the study of reproductive diseases, mice are the most commonly used animal model. However, the cost and time required to establish mouse animal models are high. The existing zebrafish model can solve this problem well. Zebrafish is an animal model with great application prospects and has lots of advantages, including high degree of genetic conservation with humans, short reproductive cycle, transparent embryos, and rapid growth, providing unique opportunities for high-throughput drug screening and identification of potential treatments. Researchers have successfully used chemical induction, physical damage, gene editing technology, etc., to induce reproductive system damage in zebrafish to study the biological processes related to its reproductive diseases. Therefore, in this review, the main models and related advantages of zebrafish in reproductive diseases are summarized, the pathological mechanisms of zebrafish as a reproductive disease model are clarified, and new perspectives and valuable insights are provided for the treatment of human reproductive diseases. The literature and data cited in the review are all from PubMed, covering important research results on zebrafish reproductive diseases in the past 10 years.

## 1 Introduction

**FIGURE 1:**
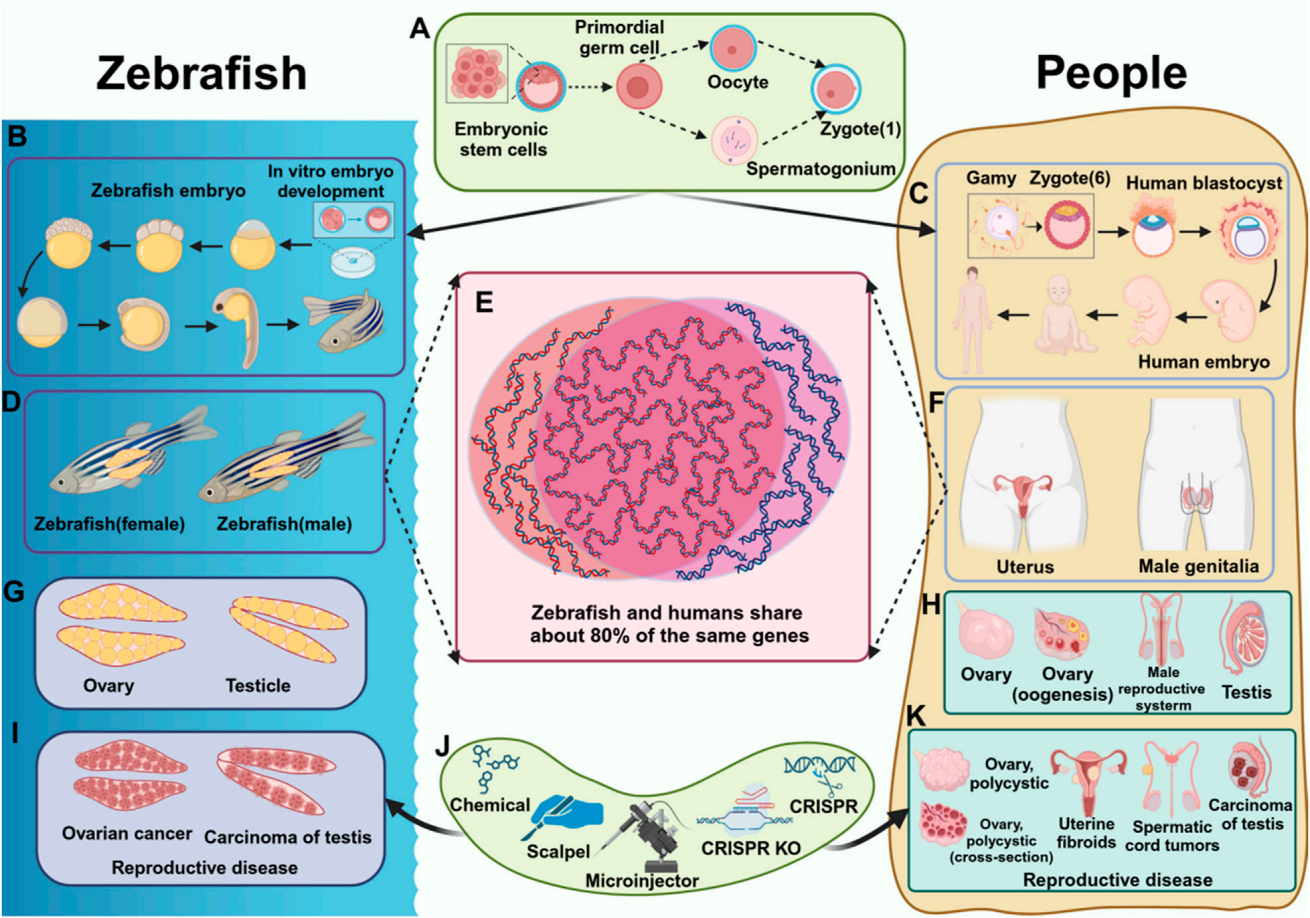
Comparison of reproductive diseases in zebrafish and humans. **(A)** The process of fertilized egg formation. **(B)** The development process of zebrafish. **(C)** The development process of humans. **(D)** The reproductive system of zebrafish (female fish, male fish). **(E)** Homologous genes in zebrafish and humans. **(F)** The reproductive system of humans. **(G)** The reproductive organs of zebrafish. **(H)** The reproductive organs of humans. **(I)** Reproductive diseases in zebrafish **(J)** Tools that cause reproductive diseases **(K)** Reproductive diseases in humans.

In recent years, zebrafish (*Danio rerio*) have become a valuable model organism in diverse research fields, including developmental biology, genomics, neuroscience, and drug development. Due to their high degree of genetic conservation with humans (Figures 1D–F), as well as similarities in tissues, organs, and genes (Figures 1G, H), zebrafish are considered an effective model for studying human diseases (Wang et al., 2021). Additionally, zebrafish offer several advantages, including a short reproductive cycle, transparent embryos, well-established genetic tools, and conserved biological processes (Callegari et al., 2023). These features enable researchers to efficiently study the formation and function of reproductive organs, the development and migration of germ cells, and the underlying mechanisms of reproductive diseases in a relatively short timeframe. While recent studies on reproductive system diseases have generated significant interest, key unresolved issues regarding the use of model organisms to investigate reproductive diseases and their mechanisms remain underexplored. Given these advantages, Zebrafish serve as an ideal model for studying reproductive diseases, facilitating a deeper understanding of the mechanisms and contributing factors involved (Sanchez and Amatruda, 2016).

Many genes related to reproduction in zebrafish have been identified and studied. At the same time, there are many genetic tools available to researchers. For example, chemical induction, physical damage, CRISPR/Cas9, and other gene editing technologies (Carver et al., 2021) (Figure 1J), which allow researchers to use these techniques to induce zebrafish germline stem cells to differentiate into specific types of cells, or cause the expression of specific genes, and construct reproductive-specific mutation models, to study genes and signaling pathways related to reproductive diseases.

This review highlights the significant advantages of using zebrafish as a model for studying reproductive diseases. Zebrafish possess remarkable reproductive capacity, and a large number of samples can be obtained in a short time. Zebrafish share a high degree of genetic homology with humans and exhibit similar drug responses, making them an excellent model for drug screening. Their ability to efficiently and accurately mimic human reproductive diseases enhances their value in studing the underlying mechanisms of these conditions. This makes zebrafish an ideal model for reproductive disease research, offering strong support for the development of therapeutic interventions aimed at improving reproductive health. Additionally, zebrafish model contribute significantly to the understanding of the pathogenesis of reproductive diseases. Various zebrafish disease models have been established, including those for gonadal development abnormalities (Shive et al., 2010; Wilson C. A. et al., 2024) (Figures 1I, K), reproductive system infections (Ventura Fernandes et al., 2022), sex hormone regulation (Yu et al., 2022; Zhai et al., 2017), and reproductive system tumors (Li et al., 2024). These models underscore the importance of zebrafish as a powerful tool for reproductive disease research, playing a crucial role in advancing the field of reproductive medicine (Geiger et al., 2008).

## 2 Early reproductive development and disease

Germline stem and progenitor cells (GSPCs) encompass primordial germ cells (PGCs) and germline stem cells (GSCs). The maintenance and differentiation of these cells are critical for sexual reproduction,as they play a key role in determining the sexual fate of germ cells (Zhang et al., 2022). The formation, maintenance, and differentiation of germline stem and progenitor cells (GSPCs) are regulated by a variety of mechanisms. Germline stem cells (GSCs) are specialized cells capable of continuous differentiation throughout the reproductive lifespan, with the ability to self-renew (Tanaka, 2016; Wang et al., 2022). During the early stages of embryogenesis, primordial germ cells (PGCs) are specified and maintained through the influence of maternal germ plasm components. The germplasm is a specialized cytoplasmic region found in the eggs and early embryos, which contains key regulatory factors that not only determine the fate of germ cells but also ensure the proper development of PGCs. These factors play an essential role throughout the entire process of reproductive development (Figures 1A–C).

Studies have demonstrated that during primordial germ cell (PGC) migration, the Tudor domain-containing protein 7 (Tdrd7) plays a crucial role in regulating the perinuclear relocalization of germplasm. Disruption of Tdrd7 leads to a reduction in PGC numbers and an increased occurrence of sex reversal (D'Orazio et al., 2021). Additionally, the germ cell nucleic acid-binding peptide (Gcna) is present in the body, where it protects them from various forms of damage, supporting a conserved mechanism that promotes the maintenance of intergenerational genome integrity (Bhargava et al., 2020). Puf-A is a member of the Pumilio RNA-binding protein family (PUF family) and plays a critical role in ribosome biogenesis. Studies have shown that Puf-A is highly expressed in zebrafish primordial germ cells (PGCs),where it is involved in their development. Knockout of Puf-A results in a significant loss of PGCs and a reduction in the motility of the remaining PGCs. Silencing of Puf-A lead to the translocation of nucleophosmin 1 (Npm1) from the nucleolus to the nucleoplasm, which in turn triggers the overactivation of *p53* in PGCs. The observed alterations in Npm1 and *p53* are similar to those seen in cancer cells, suggesting that Puf-A plays a crucial role in the regulation of PGC development and in maintaining cellular homeostasis (Ko et al., 2022).

In addition, it has been shown that the vertebrate-specific dead-end protein (Dnd1) plays a crucial role in regulating the dynamic spatial organization and function of molecules within zebrafish germ granules, Dnd1 is particularly important for maintaining the fate of germ cells (Westerich et al., 2023a; Westerich et al., 2023b). Furthermore,two novel long noncoding RNAs (IncRNAs),Inc196 and Inc172, which are highly expressed in PGCs and gonads, also contribute to the regulation of PGC development in zebrafish (Li et al., 2023). Increasing evidence shows that mRNA translation and ribosome biogenesis play a crucial regulatory role in the development and aging of germ cells. mRNA binding proteins that specifically mark germ cells, such as Pumilio, Nanos, Vasa, Yth domain-containing 2 (Ythdc2), and Dazl, are conserved during genetic development (Li et al., 2022; Mercer et al., 2021). The *vasa* gene product is particularly important for germline specification. Among them, Vasa RNA is a component of zebrafish germplasm. When the mother sends a signal, the germplasm segregation pattern will change accordingly, resulting in specific gene expression. The Vasa protein is an RNA helicase that promotes translation. It is highly expressed in germ cells during embryonic development and plays a role in the directional migration, development, and differentiation of germ cells (Hartung et al., 2014; Knaut et al., 2000).

## 3 Application and mechanisms of zebrafish reproductive diseases

### 3.1 Zebrafish as a model for mimicking human reproductive diseases

**FIGURE 2:**
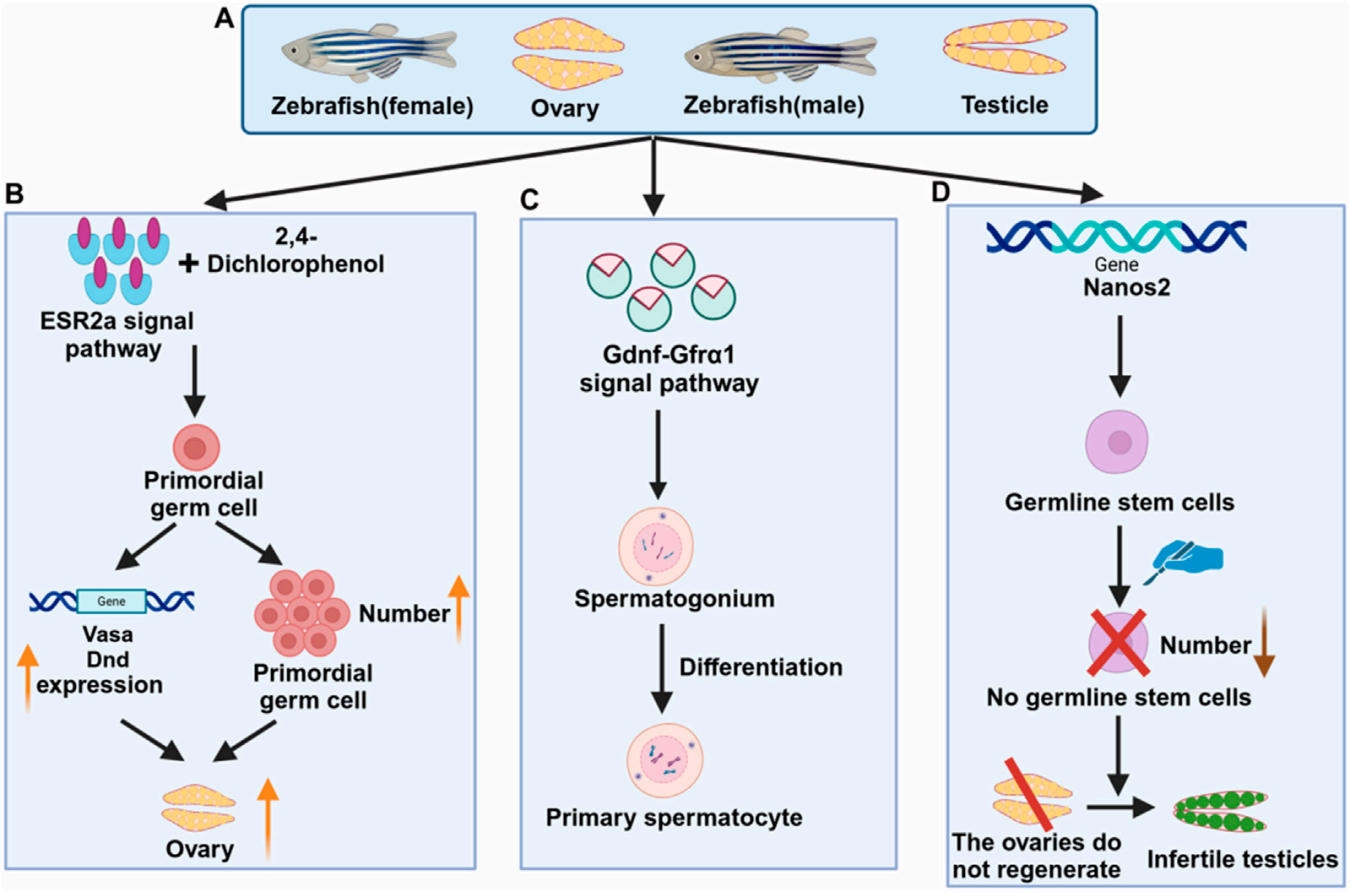
Mechanisms related to zebrafish reproductive diseases. **(A)** Female and male zebrafish and reproductive organs **(B)** Signaling pathways of female zebrafish reproductive diseases **(C)** Signaling pathways of male zebrafish reproductive diseases **(D)** Genes related to zebrafish reproductive diseases.

Zebrafish are an important part of aquatic ecology. It is low-cost, has strong reproductive capacity, and is similar to mammalian development. Therefore, zebrafish is recommended as a good experimental animal model. It is commonly used to investigate a range of biological and medical issues, including the development and function of the reproductive system (Figure 2A), as well as the onset and treatment of various diseases. Zebrafish have been studied through a variety of methods, such as evaluating its reproductive performance through reproductive behavior (zebrafish’s sperm, egg production, and hatching ability), detecting proteins through Western blot, and analyzing gene expression levels through real-time fluorescence quantitative polymerase chain reaction qPCR(Ma et al., 2020). Zebrafish reproductive disease refers to the influence of chemical induction, physical damage, gene editing technology, and other methods on the normal function of the zebrafish reproductive system, resulting in various reproductive toxicities, related diseases, or abnormal conditions. Common reproductive diseases include abnormal gonadal development, reproductive system tumors, reproductive system infections, etc. These diseases all involve the development of germ cells, the structure and function of reproductive organs, and changes in reproductive behavior.

As is well known, the CRISPR/Cas9 system is widely used as a tool for targeted genomic mutations. Recent studies have improved the CRISPR/Cas9 system in zebrafish by optimizing the Cas9 protein with zebrafish-specific codons and customizing the guide RNA. Four endogenous sites (*tyr*, *golden*, *mitfa*, and *ddx19*) and transgenic *Tg (-5.1mnx1:egfp)* were effectively targeted, with a mutagenesis efficiency of up to 99%. Various functions and phenotypes of injected zebrafish disappeared significantly. It can be seen that the CRISPR/Cas9 system is an extremely efficient gene editing technology in the zebrafish model and has great potential in biological research (Jao et al., 2013).

In addition to the diseases and injuries of the reproductive system caused by the above experiments, other reproductive diseases may be encountered, such as ambiguous sex characteristics or mixed-sex expression caused by abnormal development of reproductive organs, gonadal (ovarian or testicular) structural and functional abnormalities leading to gonadal tumors, cysts, imbalanced reproductive system development, and disordered sex hormone secretion. Therefore, studying reproductive diseases in zebrafish can provide valuable insights into the development, regeneration, and functional mechanisms of the human reproductive system, as well as the onset and progression of related diseases. This research holds significant potential for advancing our understanding of human reproductive health issues, such as infertility and gonadal tumors. This article reviews the latest technologies and tools in reproductive disease research, with a particular focus on zebrafish-related reproductive diseases and their underlying molecular mechanisms.

### 3.2 Cellular mechanisms of reproductive diseases in zebrafish

Zebrafish models had played an important role to study reproductive diseases. The reproductive system of zebrafish has many similarities with that of humans, so it can be used to study the development, function, and diseases of the reproductive system. In the study of zebrafish reproductive diseases, common cellular mechanisms include germ cell differentiation, gonadal development (Kossack et al., 2023), hormone regulation, genetic regulation, and so on. Studies have shown that insulin-like peptide 3 (INSL3) can specifically bind to the RXFP2 (G protein-coupled receptor) receptor. INSL3 has autocrine and paracrine functions in the testis and is relatively conserved during evolution (Donizetti et al., 2024). In addition, the cyclin-dependent protein kinase (CDK) inhibitor Dinaciclib combined with cisplatin (CP) can effectively improve the adverse response of most non-spermatogonia (TC) tumors to cisplatin (Rossini et al., 2024). Studies have found that thrombomodulin (TM) is involved in cell adhesion and collective cell migration *in vitro*. Full-length zebrafish TM-like protein-b (TM-b) can co-localize with actin and microtubules in epidermal blastocysts (Lee et al., 2019). In addition, zebrafish have two subtypes of vasoactive intestinal peptide (Vip), called Vipa and Vipb, of which Vipa has the highest homology with the mammalian form. Male zebrafish lacking Vipa (*vipa −/−*) show severe fertility defects, such as reduced sperm efficiency and weakened sexual motivation, and the sex ratio of offspring is biased towards females (Yu et al., 2024). The above findings demonstrate the importance of zebrafish models in the study of reproductive diseases.

The phenomenon of hormone regulation of reproductive diseases is also worthy of attention, especially arginine oxytocin (AVT). AVT can reduce the proliferation of mitotic cells and type B spermatogonia, which shows that it has important physiological significance for male reproduction (Zanardini et al., 2024). Its key marker is the Mullerian hormone (AMH/Amh), which plays a vital role in the differentiation and function of vertebrate gonads. In zebrafish, the absence of Amh results in abnormal gonadal development and function, including early germ cell accumulation and gonadal hypertrophy in both sexes. Analysis of fshb and growth differentiation factor 9 (gdf9) mutants also clarified the role of FSH in ovarian hypertrophy in young female mutants. It was demonstrated that Amh plays a dual role in maintaining gonadal homeostasis and gametogenesis in zebrafish (Zhang Z. et al., 2020).

Reproductive success relies on the proper establishment and maintenance of biological sex. In many animals, primary gonads are initially biased toward developing into ovaries, and the RNA-binding protein (RNAbp) Rbpms2 has been shown to play a critical role in determining ovarian fate in zebrafish. Targets of Rbpms2, known as rboRNAs, were identified in oocytes, and Rbpms2 was found to regulate the translation of these rboRNAs. Genetic analysis revealed that Rbpms2 promotes nucleolar expansion through the mTORC1 signaling pathway, suggesting that early germ cells exist in a dual, bipotential state. As an RNA-binding protein with multiple splice forms, Rbpms2 acts as a binary fate switch, inhibiting testicular factors while promoting oocyte development. It facilitates oocyte progression via the Rags 2 (Gator2) pathway and integrates the regulation of zebrafish oogenesis (Wilson M. L. et al., 2024). Chromosome pairing during meiosis requires the coordinated action of telomere tethering and microtubules. Telomeres are pulled toward the centrosome, forming a “zygotic chromosome bouquet.” In oocytes, “zygote cilia” were identified as crucial structures that provide a cable system for the bouquet machinery and extend into the germline cyst. Experiments in zebrafish have demonstrated that cilia anchor the centrosome, balancing the pulling of telomeres, and are essential for the formation of the bouquet and synaptic complexes, as well as for oogenesis, ovarian development, and fertility. This mechanism is conserved in both zebrafish and mice, highlighting the reproductive significance of ciliopathies and the role of cilia in regulating chromosome dynamics (Mytlis et al., 2022).

Understanding the lineage relationship between fertilized eggs and their offspring is crucial for advancing our knowledge of development and stem cell biology. While existing cell barcoding technologies in zebrafish have limited resolution, the newly developed Substitution Mutation Assisted Lineage Tracing System (SMALT) has shown promise in overcoming this limitation. SMALT has successfully been used to reconstruct the phylogenetic tree of *Drosophila melanogaster* cells and has also been applied to zebrafish, enabling the tracking of embryonic mutations. Additionally, the cell lineage tree of zebrafish fin cells was reconstructed, revealing the origins of regenerated fin cells. The analysis confirmed the stability of the germ cell pool and the early separation of germ cell and somatic cell progenitors, providing valuable insights into zebrafish development and disease research (Liu et al., 2024).

Additionally, studying the mechanisms by which hormones regulate the reproductive system and the effects of hormonal imbalances on reproductive diseases is essential. Clarifying how gene mutations or genetic variations influence reproductive system function can uncover the genetic basis of reproductive diseases. By delving into these cellular mechanisms, we can gain deeper insights into reproductive diseases, providing a scientific foundation for their prevention, diagnosis, and treatment in humans.

### 3.3 Zebrafish as a model for unraveling molecular mechanisms of human reproductive diseases

The study of the molecular mechanisms of reproductive diseases in zebrafish involves multiple aspects, including gene regulation, signal transduction pathways, environmental factors, cell biology, etc.

In terms of gene regulation, researchers can understand the mechanism of reproductive diseases by studying the expression and function of different genes in the zebrafish reproductive system. Mutations or abnormal expression of specific genes may lead to abnormal development or dysfunction of reproductive organs (Fontana et al., 2023). Large-scale transcriptome sequencing has significantly expanded the catalog of long non-coding RNAs (lncRNAs). Functional characterization of the novel, ultra-conserved lncRNA THOR has revealed its expression in the testis and various human cancers. In zebrafish, transgenic knockout of THOR induces fertilization defects and confers resistance to melanoma (Hosono et al., 2023; Mishra et al., 2021). It has been reported that the zebrafish mutant line tgct was identified as a model of spontaneous germ cell tumor (GCT) through forward genetic screening. Cloning studies have shown that interruption of type IB bone morphogenetic protein (BMP) signaling, leading to abnormal germ cell differentiation, has been identified as the root cause of zebrafish germ cell tumors (Neumann et al., 2011). There are two Nodal homologs (*ndr1* and *ndr2*) in zebrafish follicles. The mRNA levels of *ndr1*, *ndr2*, and their receptors in follicles vary at different stages. At the same time, Nodal treatment can promote zebrafish follicle growth, steroidogenesis, and oocyte maturation (Zayed et al., 2020). The role of steroids in zebrafish sex differentiation, gonadal development, and adult gonadal function can be explored using ferredoxin 1b (Fdx1b) mutant zebrafish. In adult male Fdx1b−/− mutants, androgen and cortisol concentrations are significantly reduced, spermatogenic gene expression is reduced, and the mutants primarily display female secondary sexual characteristics (Oakes et al., 2019).

Understanding the mechanisms of action of signal transduction pathways involved in regulating reproductive function in the zebrafish reproductive system can reveal the key links in the occurrence of reproductive diseases. For example, the role of hormones such as estrogen and testosterone in the reproductive system. The combination of zebrafish ESR2a signaling pathway and 2,4-dichlorophenol (2,4-DCP) leads to an increase in the number of primordial germ cells (PGCs), upregulation of PGC marker genes (*vasa* and *dnd*), and increased female levels (Hu et al., 2022) (Figure 2B). Studies have shown that glial cell line-derived neurotrophic factor (GDNF) and GDNF family receptor α1 (GFRα1) signaling pathway have a certain influence on zebrafish testes. Germ cells contribute to the formation and development of spermatogonia cysts by creating a newly available niche in the ecosystem. This process inhibits the differentiation of late spermatogonia through two distinct regulatory modes: autocrine and paracrine signaling. As a result, it promotes the maintenance of germ cell stemness (Doretto et al., 2022) (Figure 2C). Studies have shown that knockout of progesterone/nuclear progesterone receptor (nPgr) inhibits testicular development and sperma-togenesis in zebrafish. Transcriptome analysis also revealed the association of related genes, indicating that enhancing the progesterone/nPgr pathway can play a rescue role and deplete androgen signaling (Zhai et al., 2022). In addition, fertility and endocrine function rely on the precise synchronization of the hypothalamic-pituitary-gonadal (HPG) axis, with the renewal of gonadal stem cells being crucial for maintaining hormonal balance and fertility. Through genome-wide transcriptome analysis and gonadal microinjection, two GPCR regulatory loops were identified: miR430a-Sox9a in the testis and miR218a-Sox9b in the ovary. These findings offer valuable insights into gonadal differentiation and suggest potential therapeutic strategies for treating age-related gonadal diseases in humans (Guo et al., 2019; Sun et al., 2013). Follicular development is tightly regulated by both oocytes and follicular cells, with epidermal growth factor (EGF/Egf) playing a crucial role in ovarian function. By generating Egfr and Egfr mutant zebrafish, the early reproductive conditions of *EGF −/−* mutants were examined. The *egfra −/−* mutation failed follicle activation, causing a block in folliculogenesis during the transition from primary to secondary growth (PG-SG), ultimately leading to female infertility and abnormal expression of key genes. These findings highlight the essential role of EGFR signaling in early folliculogenesis (Song et al., 2021).

Studying the development, regeneration, proliferation, and differentiation of zebrafish germ cells, as well as the localization and interaction of germ cells in the reproductive system, will help us understand the occurrence and development of reproductive diseases. The study clarified that the *nanos2* gene, a conserved vertebrate germline stem cell (GSC) marker, is required for maintaining zebrafish GSCs. GSC ablation and tissue resection will lead to gonadal metastasis, that is, the ovaries cannot regenerate and turn into infertile testes (Figure 2D). It has been revealed that germline stem cells (GSCs) play a crucial role in ovarian regeneration and are essential components of the reproductive system in organisms. Studies on zebrafish germ cell regeneration have demonstrated that understanding these mechanisms can provide valuable insights into the underlying causes of human infertility,as well as offer new perspectives for studying reproductive diseases in mammals (Cao et al., 2019). RecQ helicase plays an important role in maintaining genome integrity. The creation and characterization of a zebrafish Bloom syndrome (a recessive autosomal disease) disease model showed that the zebrafish Blm mutant recapitulated the main hallmarks of the human disease and that certain functions of zebrafish Blm were essential for reproductive system development and sex differentiation. The Blm function appeared to be independent of *tp53*. The Bloom model helped us understand the occurrence of the disease and the molecular mechanisms of genome maintenance proteins in somatic DNA damage repair and fertility (Annus et al., 2022; Ignatius et al., 2018).

## 4 Useful tools for analyzing reproductive diseases in zebrafish

### 4.1 Chemicals induce germ cell damage

**FIGURE 3:**
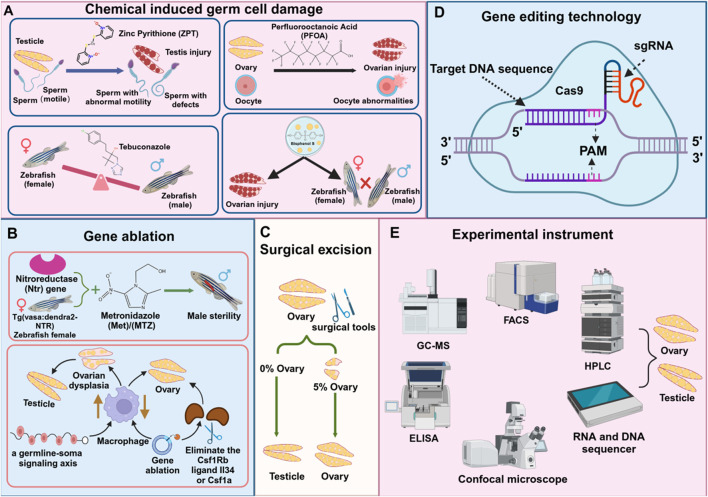
Techniques and tools for studying reproductive diseases in zebrafish. **(A)** Chemical substances damage reproductive cells **(B)** Gene ablation **(C)** Surgical injury **(D)** Gene editing technology **(E)** Instruments for detecting reproductive cells.

The impact of chemicals on the reproductive system is one of the main factors leading to reproductive diseases. These chemicals can cause germ cell damage, gonadal dysfunction, and other reproductive diseases, and involve multiple mechanisms such as chromosomal abnormalities or gene mutations caused by DNA damage, programmed death, reproductive hormone disorders, etc., which ultimately lead to germ cell dysfunction. Zebrafish (*D. rerio*), as an important model organism, provides an ideal platform for studying these mechanisms. The platinum in zinc pyrithione (ZPT), an antifouling, antibacterial, and antifungal agent, is toxic to the ecosystem. Exposure to zebrafish can induce potential mechanisms such as decreased testosterone levels, sperm deformities, reduced motility, and testicular damage (Hu et al., 2024). Exposure to the emerging persistent organic pollutant perfluorooctanoic acid (PFOA) (100 mg/L, for 15 days) in zebrafish can cause related inflammatory responses, thereby inhibiting their fertility, leading to ovarian damage and abnormal oocyte development (Zhang et al., 2023). Long-term exposure to a fungicide, tebuconazole, in zebrafish, can cause an imbalance in the male-female ratio, seriously endangering the reproductive health of fish (Qiao et al., 2023). Studies have shown that bisphenol S (BPS) poses a potential threat to the reproductive system. Exposure to BPS in adult zebrafish can cause ovarian function decline, hinder oocyte maturation, reduce mating ability, and cause the survival behavior of offspring to deviate from normal (Wang et al., 2020) (Figure 3A). Therefore, analyzing chemical-induced germ cell damage can help us further understand the development process of human reproductive diseases.

### 4.2 Gene ablation

Gene ablation technology is widely used to study the mechanism of reproductive system development and dysfunction, inhibit or reduce the expression level of specific genes in reproductive diseases, and ablate related target cells (Cao et al., 2021; Kumar and Elkouby, 2023). Researchers can further elucidate the mechanisms underlying reproductive development abnormalities by simulating the onset of reproductive diseases, thereby providing valuable insights for prevention and treatment strategies. Studies have demonstrated that the *Escherichia coli* nitroreductase (*ntr*) gene can convert non-toxic prodrugs, such as metronidazole (MTZ), into cytotoxic compounds. When zebrafish expressing the *ntr* gene were treated with MTZ, the target cells in the testis were selectively ablated, leading to induced male infertility (Hsu et al., 2010). After a period of MTZ ablation, *Tg (vasa:Dendra2-NTR-vasa 3'UTR) cq41* transgenic females were constructed. After a period of MTZ ablation, no early germ cells appeared in the ovaries. Even if mature eggs were produced, they eventually recovered to infertile males. It was revealed that genetic ablation of GSCs would lead to ovarian regeneration failure (Cao et al., 2019). Studies have established a causal link between macrophage activation and ovarian failure using a zebrafish model. In vertebrates, the loss of BMP15, a growth factor essential for ovarian development and maturation, disrupts oocyte formation and initiates a cascade of downstream effects. In humans, ovarian insufficiency can be caused by autoimmune and genetic factors, including mutations in BMP15. This forms a signaling axis in germ cells, triggered by the loss of Bmp15, which activates macrophages, leading to ovarian failure and masculinization. Genetic ablation of macrophages of the Csf1Rb ligands, such as Il34 or Csf1a, can delay or even prevent premature oocyte aging and sex reversal (Bravo et al., 2023) (Figure 3B).

### 4.3 Physical damage: surgical resection

Surgical resection refers to the removal of specific tissues and organs through surgical methods to simulate the development of the disease, so as to study its pathological changes, pathogenesis, and corresponding treatment methods. This technology is widely used in disease modeling and for the removal of various organs, including the reproductive system, liver, and heart. Studies have shown that when a large portion of the zebrafish ovary is removed, the remaining approximately 5% of ovarian tissue can fully regenerate in a short period, restoring the original reproductive function. This demonstrates the remarkable regenerative ability of zebrafish ovaries. However, if the entire ovary is removed, regeneration does not occur, and the fish permanently lose the ability to lay eggs and reproduce naturally, ultimately transforming into sterile testes (Cao et al., 2019) (Figure 3C).

### 4.4 Gene editing technology CRISPR/Cas9 system

Clustered Regularly Interspaced Short Palindromic Repeats (CRISPR/Cas9) is a revolutionary gene editing technology used to accurately and efficiently edit the genome of an organism. It is applied in the field of life sciences and is an effective potential therapeutic strategy for treating genetic diseases (Chang et al., 2020) (Figure 3D). Compared with traditional gene editing technologies such as zinc finger nucleases (ZFNs) and transcription activator-like effector nucleases (TALENs), CRISPR/Cas9 has significant advantages. It does not require the design of complex proteins for specific target genes, making it more universal and economical (Shimizu et al., 2023). In 2012, a research team led by scientists such as Jennifer Doudna and Emmanuelle Charpentier successfully developed a powerful gene-editing tool based on this discovery. Their work unveiled the potential and mechanism of the CRISPR/Cas9 system, demonstrating that it could be used for genome editing and applied to human cells.

The CRISPR/Cas9 system uses single-stranded RNA (sgRNA) to guide the Cas9 enzyme into zebrafish embryos. Successful genetic edits are then identified through PCR, restriction fragment length polymorphism (RFLP) analysis, or sequencing, enabling targeted genetic modifications. This technology allows for precise editing of zebrafish genes, facilitating a deeper understanding of their development, reproduction, behavior, and immune system. It also holds great potential for advancing drug development, personalized medicine, gene therapies, and creating more accurate animal models for human diseases (Ma and Liu, 2015). There is literature that describes the construction of a zebrafish model with a thyroid hormone receptor α gene (*thrab*) mutation using CRISPR/Cas9 technology and explores the role of thyroid hormone signaling in female zebrafish reproduction. It has been demonstrated that thyroid hormone signaling has a profound effect on female reproductive function through the loss of the *thrab* receptor, including symptoms such as impaired oviduct development, spawning failure, female infertility, ovarian hypertrophy, follicular atresia, and ovarian tissue degeneration (Ai et al., 2024).

Studies have shown that the *cntd1* gene of zebrafish was knocked out by CRISPR/Cas9 technology, revealing the molecular mechanism by which *cntd1* gene defects lead to impaired meiotic crossover formation, resulting in the production of unreduced eggs and the formation of polyploidy (Ou et al., 2024). When CRISPR/Cas9 technology is used to knock out genes in zebrafish, there is a problem of low survival rate of knocked-out embryonic lethal genes. To overcome this problem, a new strategy combining CRISPR/Cas9-mediated gene knockout with primordial germ cell (PGC) transplantation (PGCT) was developed. The CRISPR/Cas9-targeted PGCT approach was optimized to generate maternal-zygotic (MZ) mutants of *tcf7l1a*, *pou5f3*, and *chd*. The phenotypes of the MZ mutants of *tcf7l1a* and *chd* were characterized, and an efficient method for generating MZ mutants of embryonic lethal genes in zebrafish was proposed. This approach can also be applied to accelerate genome editing in commercial fish species (Zhang F. et al., 2020).

A potential problem with CRISPR/Cas9 gene editing technology is off-target effects (Höijer et al., 2022), that is, editing occurs at locations other than the target gene, resulting in gene changes. However, off-target effects can be further reduced by selecting appropriate sgRNA design, selecting Cas9 variants with higher specificity, and introducing modified Cas9 proteins (Cui et al., 2020; Naito et al., 2015). It has been reported that hei-tag (high efficiency-tag) in the form of mRNA can effectively improve the activity of CRISPR/Cas gene editing technology. Adding hei-tag (myc-tag coupled to an optimized NLS via a flexible connector) to Cas9 or C-to-T (cytosine-thymine) base editors can significantly improve targeting efficiency (Thumberger et al., 2022). Recent studies have found that inserting a targeted mutagenesis system CRIMP and a plasmid toolkit CRIMPkit containing 24 plasmid vectors into CRISPR/Cas9 can efficiently obtain null mutation alleles without genetic compensation (Miles et al., 2024). In summary, chemical-induced injury, gene ablation, surgical resection, and CRISPR/Cas9 gene editing technology have played an important role in the study of zebrafish reproductive diseases. These tools provide valuable resources for revealing the mechanisms of reproductive diseases and finding treatments.

### 4.5 Monitoring and analysis of environmental pollutants

#### 4.5.1 Environmental sample analysis and exposure experiments

By analyzing the content of pollutants in water, sediments, and biological samples, the pollution level of chemicals in the environment and their potential impact on the reproductive system of zebrafish can be evaluated. High-performance liquid chromatography (HPLC), gas chromatography-mass spectrometry (GC-MS) (Lee et al., 2020), and liquid chromatography-mass spectrometry (LC-MS/MS) can be used to detect and quantify harmful substances in environmental samples (Fanti et al., 2020) (Figure 3E). By simulating the exposure to pollutants in the natural environment, the reproductive health of zebrafish at different concentrations and exposure times is studied. This method helps to understand the long-term effects of environmental pollution on the reproductive system of aquatic organisms.

### 4.6 Advanced imaging technology

#### 4.6.1 *In vivo* imaging and high-resolution microscopy

By using confocal microscopy, multiphoton microscopy, and other technologies, we can observe the structural and functional changes of zebrafish germ cells and tissues in normal and abnormal states in real-time (Zhang Z. et al., 2021). *In vivo* imaging can provide dynamic and physiologically relevant information. Super-resolution microscopy (such as STORM, and PALM) can be used to observe microstructural changes inside germ cells, helping to understand the effects of chemicals or genetic manipulation on cell ultrastructure (Siegerist et al., 2018).

### 4.7 Behavioral studies

By observing and recording the mating behavior, reproductive success rate, and offspring survival rate of zebrafish, the effects of chemicals or genetic manipulation on their reproductive behavior can be evaluated. Behavioral studies can provide a comprehensive assessment of reproductive health.

## 5 Zebrafish reproductive disease models

### 5.1 Gonadal disease models

The gonadal development process of zebrafish is similar to that of mammals, and the reproductive cycle is short, making it easy to conduct experiments and an ideal model for studying reproductive diseases. The occurrence of zebrafish gonadal diseases is relatively complex and is regulated by genetic, hormonal, and environmental factors. Among them, natural sex hormones synthesized by gonadal interstitial cells also affect the function of reproductive organs and the development of secondary sexual characteristics (Ruan et al., 2024). Zebrafish gonadal diseases may lead to endocrine system disorders, abnormal ovulation, poor sperm quality, and tumors such as ovarian cancer and testicular cancer. Recent studies have identified zebrafish MitoPLD, a member of the phospholipase D superfamily (also knownas *pld6*), as a novel lineage-specific gene involved in mitochondrial fusion. MitoPLD plays a critical role in cell differentiation and reproductive development. Disruption of *pld6* expression leads to embryonic infertility and masculinization (Zhang et al., 2022). DNA methylation is a key regulator of fertility (Zhang Y. et al., 2021), with mutations in DNA methyltransferase (DNMT) leading to male infertility in mice and alterations in spermatogonia stem cells (SSCs) populations (Dura et al., 2022). Therefore, researchers use zebrafish disease models to study the occurrence of gonad-related diseases, which helps us to gain a deeper understanding of the relevant mechanisms of human reproductive diseases and provides important clues and references for the treatment of reproductive diseases.

#### 5.1.1 Testicular disease models

**FIGURE 4:**
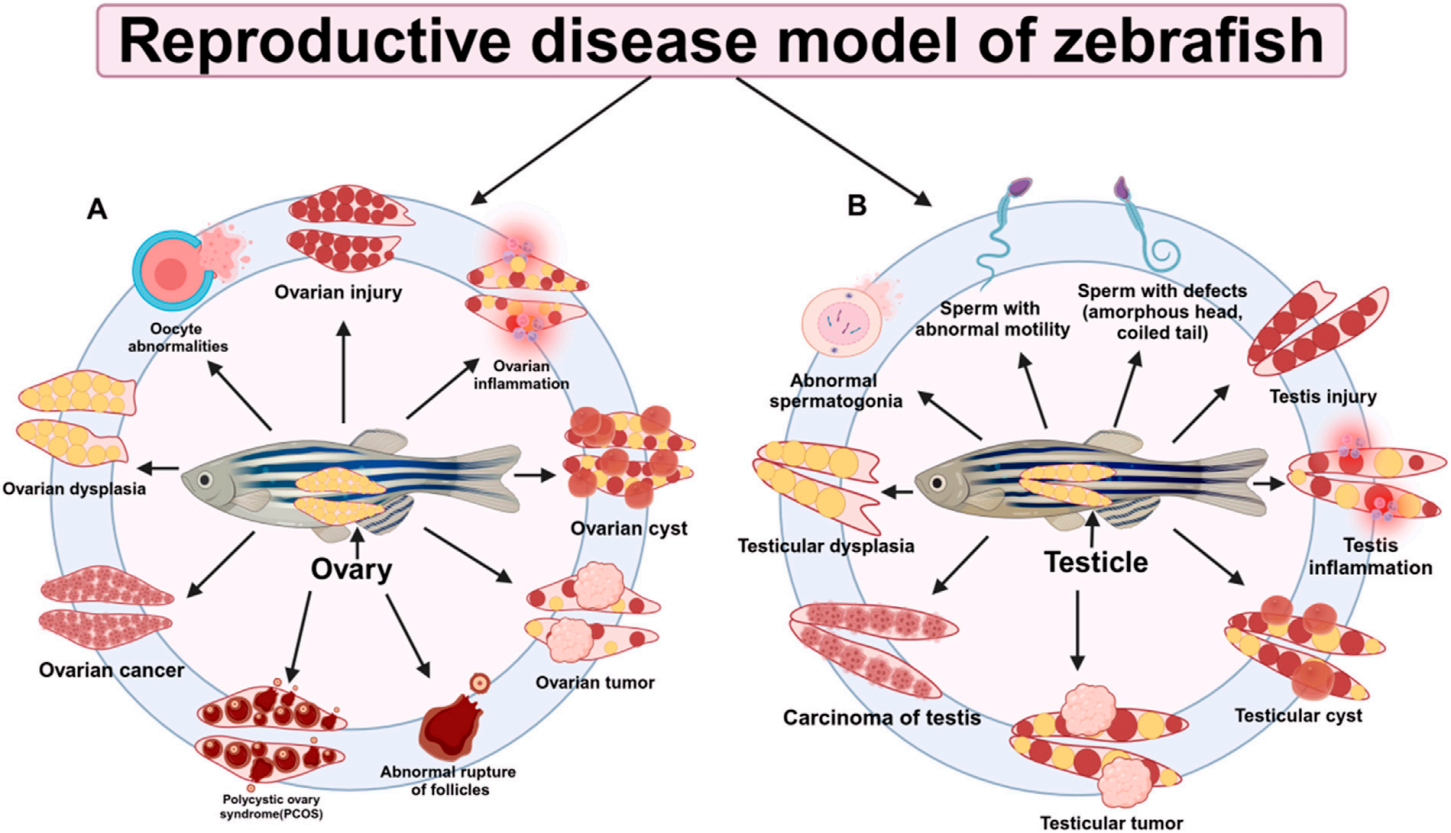
Research on reproductive disease models in zebrafish. **(A)** Zebrafish ovary-related diseases **(B)** Zebrafish testis-related diseases.

Testicular interstitial cells mainly produce testosterone, which exerts its physiological function by binding to androgen receptors (AR) and activating AR. Therefore, it is widely used to evaluate the interference of environmental pollutants on androgens at the cellular level. Sperm is the functional gamete of males, and its quality is closely related to the success rate of fertilization and the development of offspring. Taking advantage of *in vitro* culture, zebrafish sperm not only provides a good model for rapid screening of male reproductive toxicity but also for paternal genetic toxicity (Fu et al., 2024). By performing single-cell transcriptome sequencing on zebrafish testes, new spermatogenesis marker genes and stronger paracrine interactions between Leydig cells and germ cells were discovered, providing an important resource for studying zebrafish spermatogenesis (Qian et al., 2022). In addition, it was found that about 50% of infertility is caused by male factors, such as oligospermia and azoospermia (OAT),as summarized in Table 1. Exome sequencing identified pathogenic variants in X-linked germ cell nuclease (*gcna*) in patients with azoospermia. The prevalence of pathogenic variants in this gene in infertile men was evaluated, and the expression of *gcna* in testicular biopsies of patients was measured. It was found that spermatogenesis was arrested in patients with azoospermia. *gcna* is very important for genome integrity and its deletion can lead to azoospermia, severe oligospermia, low fertilization rate, and infertility (Arafat et al., 2021) (Figure 4B).

**TABLE 1:** Gene-edited zebrafish reproductive disease models and corresponding human diseases.

Gene editing	Species/Sex	Zebrafish disease models	Corresponding human diseases	Characteristics/Conclusion	References
*gcna*	Zebrafish/male	Abnormal Testicular Development Model	Oligo-astheno-terato-zoospermia (OAT)	Spermatogenesis stagnation	Arafat et al. (2021)
*tox3* and *denndla*	Zebrafish/female	Model of abnormal ovarian development	Polycystic ovary syndrome (PCOS)	Abnormal follicular development	Sudhakaran et al. (2023)
*oxr1a*	Zebrafish/female	Model of abnormal ovarian development	Premature ovarian failure (POF)	the ovarian dysfunction and defective oocyte development	Xu, Mao, Nie and Li (2023)
*tp53*、 b*rca1*、and b*rca2*	Zebrafish/female	Reproductive system tumors and infection models	Epithelial ovarian cancers (EOC)	Abdominal distension, endometriosis	Ciucci, Buttarelli, Fagotti, Scambia and Gallo (2022)
Angiopoietin-like 4 (*angptl4*)	Zebrafish/female	Reproductive system tumors and infection models	Ovarian cancer (OC)	Increased vascularization	Li et al. (2024)
Cilia-microtubule genes (*cmg*)	Zebrafish/male	Reproductive system tumors and infection model	Testicular germ cell tumour (TGCT)	Testicular enlargement and scrotal swelling	Litchfield et al. (2016)

#### 5.1.2 Ovarian disease models

Ovarian development is a highly complex process regulated by multiple factors. Advances in targeted gene-editing technologies have uncovered numerous regulatory genes and mechanisms involved in this process. These breakthroughs highlight the utility of zebrafish as an ideal model system for studying ovarian development. The ability to manipulate specific genes in zebrafish provides researchers with a powerful tool to dissect the intricate molecular pathways governing ovarian formation and function, offering valuable insights into reproductive biology and potential therapeutic targets (Li and Ge, 2020). Studies using female zebrafish as a model have shown that low doses of acetochlor can promote ovarian development, while high doses can disrupt ovarian development (Zhang Y. et al., 2020). Other studies have successfully constructed a zebrafish polycystic ovary syndrome (PCOS) model through genetic manipulation and drug induction. This model presents phenotypic and biochemical characteristics similar to human PCOS, providing a new perspective for the study of the pathogenesis and treatment of PCOS (Nehru et al., 2024; Sudhakaran et al., 2022; Zhang et al., 2024).

In addition, through experiments on CHO cells and PCOS zebrafish models, it was found that Nimbin analog N2 can alleviate high testosterone-induced oxidative stress, restore damaged follicle maturation, and change the expression of PCOS susceptibility genes *tox3* and *dennd1a*,opening up new ideas for the treatment of PCOS (Sudhakaran et al., 2023), as summarized in Table 1. Other studies have revealed the intergenerational transmission mechanism of antibiotics in fish and their toxic effects on reproductive development through two-generation exposure experiments on zebrafish, providing a new perspective for assessing the environmental risks of antibiotics to fish (Xu et al., 2024). The premature ovarian failure (POF) model is also a typical model, which refers to ovarian failure, often manifested by symptoms such as decreased estrogen levels and oocyte development defects. Studies have found that antioxidant 1a (*oxr1a*), an ortholog of mammalian *oxr1*, has a protective effect on female zebrafish oocytes. Knocking out *oxr1a* exacerbates the occurrence of POF phenotypes and leads to poor oocyte quality. In addition, *oxr1a* participates in the oxidative stress process by regulating the mRNA expression levels of antioxidant enzymes Cat and Sod1 and reduces mitochondrial oxidative damage through antioxidant treatment to ensure female fertility, thereby deeply understanding the mechanism of reproductive diseases (Xu et al., 2023) (Figure 4A).

### 5.2 Sex hormone regulation model

Sex hormones are critical regulators of the development and function of the reproductive system. By leveraging advanced gene-editing technologies, researchers can knock out or overexpress genes associated with sex hormones to investigate their specific roles and underlying molecular mechanisms (Shu et al., 2020). This approach provides valuable insights into how sex hormones regulate reproductive processes (Salanga et al., 2020). Additionally, zebrafish serve as an effective model for drug screening aimed at treating sex hormone-related disorders, offering a platform for identifying novel therapeutic targets for clinical applications. Key biomarkers, such as an increased proportion of female zebrafish, elevated levels of vitellogenin (Vtg), and altered expression of genes involved in sex hormone synthesis, further enhance the utility of zebrafish models in this context. These findings collectively contribute to the development of innovative strategies for understanding and managing diseases influenced by sex hormone dysregulation (Hu et al., 2021).

### 5.3 Reproductive system tumors and infection models

Reproductive system tumors, as common diseases, pose a significant threat to human health. Zebrafish, both at the embryonic and adult stages, offer versatile platforms for drug screening, with the added convenience of administering drugs directly into embryonic water. Notably, approximately 70% of human genes have at least one zebrafish ortholog, enabling the construction of cancer models that closely mimic human malignancies through genetic manipulation (Howe et al., 2013). To address limitations inherent in genetic engineering models—such as challenges in controlling every stage of disease progression—innovative techniques involving the transplantation of cancer cells into zebrafish embryos or adult fish have been developed. These approaches provide a complementary strategy for studying tumor biology and screening therapeutic agents, enhancing the utility of zebrafish as a model organism in cancer research (Kirchberger et al., 2017).

More than 90% of ovarian malignancies are classified as epithelial ovarian cancer (EOC) (Ciucci et al., 2022), as summarized in Table 1, which is characterized by increased vascularization and aggressive tumor growth. Among these, ovarian cancer (OC) is one of the most common and severe forms of malignant tumors, as summarized in Table 1. The high prevalence and complexity of EOC underscore the urgent need for effective diagnostic and therapeutic strategies to improve patient outcomes. Among them, angiopoietin-like 4 (*angptl4*) plays a key role in tumorigenesis. *angptl4* accelerates the carcinogenesis of ovarian serous cystadenocarcinoma and angiogenesis in the tumor microenvironment by activating the JAK2/STAT3 pathway and interacting with ESM1(Li et al., 2024) (Figure 4A). Testicular germ cell tumor (TGCT) is a common cancer in young men (Figure 4B). Whole exome sequencing of multiple TGCT cases and controls revealed that 8.7% of TGCT families had rare destructive mutations in the ciliary microtubule gene (*cmg*), compared with 0.5% in the control group, as shown in Table 1. The most significantly mutated *cmg* was *dnaaf1*, whose expression was lost in carrier tumors. The dnaaf1hu255h (+/−) zebrafish model showed that *dnaaf1* mutations were the cause of TGCT, indicating that *cmgs* are susceptibility genes for cancer (Litchfield et al., 2016), as summarized in Table 1.

Zebrafish models are used to study human germ cell tumors and to elucidate the conserved genetic program of germ cell tumor development. Experiments were performed to determine the germ cell origin of the tumor and to demonstrate that zebrafish carry haploinsufficiency of the BMP family receptor *bmpr1bb* as a mechanism of tumor formation. Comparison of gene expression profiles of human and zebrafish germ cell tumors revealed unique overlapping features. JUP was found to be a potential driver gene (Sanchez et al., 2019). Through the study of zebrafish reproductive system tumors and infection models, we can gain a deeper understanding of the occurrence of human cancer genes, provide an important theoretical basis for overcoming the problem of cancer, and bring new hope and direction to the development of human health.

## 6 Outlook

As an important model organism, zebrafish have a high degree of genetic homology with humans and have unique advantages in studying reproductive diseases, bringing many opportunities to this field. The following is an outlook on its future as a model system for studying reproductive diseases:

First, high-throughput drug screening has a promising future. Zebrafish have a high fertility rate, fast embryonic development, and *in vitro* fertilization, which is conducive to large-scale drug screening. Drug treatment can quickly evaluate its effects on the reproductive system and screen out potential therapeutic drugs. Second, it is expected to help the development of personalized medicine. Advances in gene editing technology have made it possible to construct transgenic models of human diseases, simulate specific genetic variations, and provide patients with more precise treatment options. At the same time, environmental factor assessment is crucial. The impact of the environment on reproductive health has gradually attracted attention. Through the study of zebrafish exposed to pollutants, its potential risks to human reproductive health can be assessed.

Integrated research approaches are increasingly recognized as a valuable trend in advancing scientific understanding. By incorporating multiple model systems, such as zebrafish, mammals, and cell-based models, researchers can generate complementary data. This comparative and integrative strategy allows for a more holistic exploration of the complex mechanisms underlying reproductive diseases, bridging knowledge gaps and enhancing the validity of conclusions. Finally, the potential for translational applications offers exciting prospects. Research findings could pave the way for clinical advancements. For instance, drugs or therapies initially identified through zebrafish models could be further validated in clinical trials, providing innovative approaches and strategies for the treatment of reproductive diseases. This seamless integration of preclinical research with clinical practice underscores the value of model organisms in bridging basic science and applied medicine.

However, the zebrafish model has limitations, such as physiological differences and drug dose conversion problems. And with the development of technology, other model systems are also improving. Future research needs to comprehensively consider its advantages and limitations, and combine multiple methods to promote the in-depth development of reproductive disease research.
